# Expanded dataset of mechanical properties and observed phases of multi-principal element alloys

**DOI:** 10.1038/s41597-020-00768-9

**Published:** 2020-12-08

**Authors:** Christopher K. H. Borg, Carolina Frey, Jasper Moh, Tresa M. Pollock, Stéphane Gorsse, Daniel B. Miracle, Oleg N. Senkov, Bryce Meredig, James E. Saal

**Affiliations:** 1 Citrine Informatics, Redwood City, CA United States; 2 Materials Department, University of California at Santa Barbara, Santa Barbara, California 93101 USA; 3 CNRS, Univ. Bordeaux, Bordeaux INP, ICMCB, UMR 5026, F-33600 Pessac, France; 4 Air Force Research Laboratory, Materials and Manufacturing Directorate, Wright-Patterson AFB, OH 45433 USA

**Keywords:** Metals and alloys, Mechanical properties

## Abstract

This data article presents a compilation of mechanical properties of 630 multi-principal element alloys (MPEAs). Built upon recently published MPEA databases, this article includes updated records from previous reviews (with minor error corrections) along with new data from articles that were published since 2019. The extracted properties include reported composition, processing method, microstructure, density, hardness, yield strength, ultimate tensile strength (or maximum compression strength), elongation (or maximum compression strain), and Young’s modulus. Additionally, descriptors (e.g. grain size) not included in previous reviews were also extracted for articles that reported them. The database is hosted and continually updated on an open data platform, Citrination. To promote interpretation, some data are graphically presented.

## Abstract (summary)

**Table:** 

Measurement(s)	alloy • oxygen content • carbon content • nitrogen content • yield strength • elongation • ultimate strength • hardness • density • grain size • observed phases
Technology Type(s)	digital curation
Factor Type(s)	alloy processing method • test temperature • test type • alloy composition

Machine-accessible metadata file describing the reported data: 10.6084/m9.figshare.12804137

## Background & Summary

Traditional engineering alloys consist of a single principal element (e.g., Fe in steels and Ni in superalloys) and one or more solute elements present in much lower concentrations than the principal element. In contrast, multi-principal element alloys (MPEAs), also called complex concentrated alloys (CCAs), are a class of alloys where no single element dominates the composition and 3 or more principal elements are present in significant amounts. The term high entropy alloy (HEA) is often used to describe MPEAs with 5 or more principal elements and medium entropy alloy typically describes MPEAs with 3 or 4 principal elements. These alloys exhibit unique and extensively tunable properties compared to traditional single principal element alloys^1–11^.

A primary driver of interest in MPEAs is the significant expansion in compositional design space for new alloy development made available compared to traditional alloys^12^. Assuming a palette of 30 elements to choose from, there are approximately 143,000 potential 5-component systems and 594,000 potential 6-component systems to explore, with countless compositions within each system to synthesize and characterize, often with unknown processing routes. This large design space presents a challenge, since examining each system experimentally is prohibitively expensive. As such, there has been recent interest in employing computational and data-driven methods to accelerate exploration of MPEA systems and identify promising candidates for experimental study^13,14^.

Since the approach for MPEA design was defined in 2004^1,2^, there has been a growing body of work in the literature exploring these systems experimentally, with a focus on mechanical properties. An accurate accounting of high quality data from these studies is necessary to aid in further MPEA development, such as identifying gaps in design space, training machine learning models, flagging of outliers, etc. Given the large interest in this class of alloys, data on new systems are rapidly being published, necessitating frequent database updates to maintain relevancy. The updated MPEA mechanical properties database presented here combines data from previous reviews, makes corrections to data, and adds new data from articles published in 2019. The complete database will be hosted online in conjunction with a template to ensure routine updating and public availability of the database.

## Methods

### Extraction from literature

Two previous reviews of MPEA mechanical properties from 2018^15,16^ were used to populate the initial database. When combined, these reviews contained data on 296 unique MPEA compositions (614 composition-property combinations). The additional data extracted for this study included 334 unique MPEA compositions (931 composition-property combinations), more than doubling the existing data. During extraction and digitization of the initial database, various typos and extraction errors were identified and corrected. Once digitized, the initial database was combined with the newly extracted data and put into single spreadsheet, as demonstrated in Table 1.

**Table 1:** The 25 MPEAs in the database with the highest yield strength (at room temperature), illustrating how a subset of the data are stored in each field.

DOI	FORMULA	YS (MPa)	UTS (MPa)	Elongation (%)
10.1063/1.2734517	Al1 Co1 Cr1 Fe1 Ni1 Ti0.5	2260	3140	24
10.1063/1.2734517	Al0.667 Co0.667 Cr0.667 Fe0.667 Ni0.667 Ti1	2220	2720	7
10.1016/j.actamat.2016.01.018	Hf1 Nb1 Ta1 Zr1	2100	2200	4
10.1007/s11837-014-1066-0	Al0.333 Nb0.667 Ta0.533 Ti1 V0.133 Zr0.667	2035	2105	5
10.1016/j.matdes.2013.04.061	Al0.7 Co0.3 Cr1 Fe1 Ni1	2033	2635	8
10.1007/s11837-014-1066-0	Al1 Mo0.5 Nb1 Ta0.5 Ti1 Zr1	2000	2368	10
10.1007/s11837-014-1066-0	Al0.214 Nb0.714 Ta0.714 Ti1 Zr0.929	1965	2054	5
10.1007/s11837-014-1066-0	Al0.214 Nb0.714 Ta0.571 Ti1 V0.143 Zr0.929	1965	2061	5
10.1016/j.msea.2006.11.049	Al0.125 Co1 Cr1 Cu1 Fe1 Mn1 Ni1 Ti1 V1	1862	2431	1
10.1063/1.2734517	Al1 Co1 Cr1 Fe1 Ni1 Ti1	1860	2580	11
10.1007/s11837-014-1066-0	Al0.4 Hf0.6 Nb1 Ta1 Ti1 Zr1	1841	2269	10
10.1007/s11837-012-0366-5	Mo1 Nb1 Ti1 V1 Zr1	1786	3828	26
10.1007/s11837-012-0366-5	Mo1 Nb1 Ti1 V0.25 Zr1	1776	3893	30
10.1007/s11837-012-0366-5	Mo0.667 Nb0.667 Ti0.667 V1 Zr0.667	1735	3300	20
10.1016/j.matdes.2015.05.019	Hf1 Mo1 Nb1 Ti1 Zr1	1719	1803	10
10.1007/s11837-012-0366-5	Mo1 Nb1 Ti1 V0.75 Zr1	1708	3929	29
10.1007/s11837-012-0366-5	Mo1 Nb1 Ti1 V0.5 Zr1	1647	3307	28
10.3390/e16020870	Al0.5 Mo1 Nb1 Ti1 V1	1625	1800	11
10.1016/j.intermet.2015.03.013	Hf1 Mo1 Ta1 Ti1 Zr1	1600	1743	4
10.1016/j.msea.2011.09.033	Cr1 Mo0.5 Nb1 Ta0.5 Ti1 Zr1	1595	2046	5
10.1007/s11837-012-0366-5	Mo1 Nb1 Ti1 Zr1	1592	3450	34
10.4028/www.scientific.net/MSF.849.76	Hf1 Nb1 Si0.5 Ti1 V1 Zr1	1540	1643	17
10.1007/s11837-012-0366-5	Mo0.5 Nb0.5 Ti0.5 V1 Zr0.5	1538	3176	23
10.1016/j.msea.2016.0710.102	Nb1 Ta1 V1 W1	1530	1700	12
10.3390/e18050189	Mo1 Nb1 Ta1 V1	1525	2400	21

For alloys with multiple reports, the report resulting in the highest yield strength is shown. Many properties are not shown in this view.

**Fig. 1:**
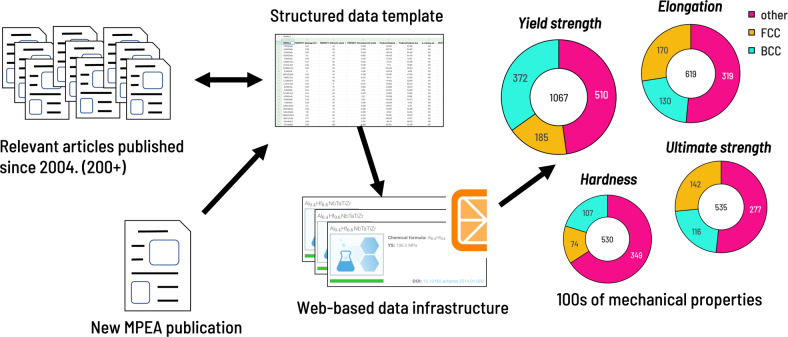
The database generation workflow. Records are first extracted from various publications and input into a defined template format. Post-processing tools are used to identify outliers or erroneous data points. A detailed review of the number of records and properties contained in the resultant database is presented in Table 2.

To identify new sources of MPEA data, a keyword search for “high entropy alloy” was conducted on Web of Science (query performed October 2019) and responses were filtered for articles published in 2019. From this query, 136 articles were identified as potentially viable sources of experimental MPEA mechanical property data (i.e. articles reporting single and multiphase materials with a minimum of three elements). Defined in detail in the Data Records section, relevant mechanical property data were extracted from plots, tables, and text and input into a tabular format. To extract data from plots, webplotdigitizer^17^ was employed. The newly extracted data were combined with the previously digitized data to complete the database. A high level overview of the extraction workflow is provided in Fig. 1.

### Data from future publications

For any data that are relevant, but not present in the current review, researchers are encouraged to make their own contributions. Using the template provided on GitHub^18^ data extraction and digitization can be performed by many groups asynchronously. This template is formatted such that data can be easily uploaded to Citrination, an online platform for materials data^19^. Upon notification, any data added to the database on Citrination will be verified for integrity by the authors. Researchers are also encouraged to upload their data to other open data resources and contact the authors directly for integration with the MPEA database.

## Data Records

The database contains 1545 records from 265 articles. An individual record is defined as having a unique composition, property, temperature, reference combination. For example, if two articles measured the yield strength of HfNbTaTiZr at five temperatures, the number of records extracted is ten. On a per record basis, this database presents a > 100% improvement in the amount of available data when compared to the data presented in the 2018 reviews.

The data in the database are extracted to best represent the data made available by the authors. Often, not all properties in the database are reported for every record. For example, despite the importance of grain size and interstitial contents on properties, particularly for refractory MPEAs, these features are missing from many articles. The data are made available on Figshare^20^ and in various tabular formats on the project GitHub^18^. The data have also been digitized into Physical Information File (PIF) records, an open-source json-based schema for materials data^21^. PIF records are hosted on Citrination (https://citrination.com/datasets/190954) to provide easy access for data visualizations and machine learning. Each data source will be updated continuously as more data are extracted.

The database records consist of the following fields, as available:

- **Alloy composition**: Normalized and alphabetized nominal alloy composition, in atomic percent. Validation and alphabetization were performed using the Pymatgen Composition module^22^.
- **Microstructure**: The experimentally observed phases (e.g. FCC, BCC, B2). Any phases that were not BCC, FCC, HCP, L12, B2, or Laves were labeled as “Sec. = secondary” or “Other”.
- **Processing method**: The conditions under which the alloy was synthesized. CAST = as-cast or directional casting. POWDER = gas atomization, mechanical alloying, sintering, spark plasma sintering, or vacuum hot pressing. WROUGHT = cold-rolled, hot-rolled, or hot-forged. ANNEAL = annealed, homogenized, or aged. OTHER = additive manufacturing, hot isostatic pressing, or severe plastic deformation.
- **Grain size (*μ*m)**: The average grain size of the alloy.
- **Exp. Density (g/cm**^**3**^**)**: Experimentally reported density.
- **Calculated Density (g/cm**^**3**^**)**: Density estimated using the rule of mixtures (ROM): $\rho =\Sigma {x}_{i}{M}_{i}/\Sigma {x}_{i}{V}_{i}$ where *x*_*i*_, *M*_*i*_, *V*_*i*_ are the atomic fraction, molar mass and molar volume of the element *i*. Elemental density values were obtained via Pymatgen^22^.
- **Type of test**: Indicator for whether mechanical testing was performed under tension (T) or compression (C).
- **Test temperature (°C)**: Temperature at which mechanical testing was performed.
- **YS (MPa)**: Measured yield strength.
- **UTS (MPa)**: Measured ultimate tensile strength (for tensile tests) or maximum compression strength (for compression tests).
- **HV**: Experimentally reported Vickers hardness.
- **Elongation (%)**: Measured elongation at failure or maximum reported compression strain.
- **Elongation plastic (%)**: Measured plastic elongation or plastic compression strain.
- **Exp. Young’s modulus (GPa)**: The experimental Young’s modulus, when reported.
- **Calculated Young’s modulus (GPa)** Young’s modulus calculated using the rule of mixtures (ROM) for single phase solid solutions only: $E=\Sigma {x}_{i}{V}_{i}{E}_{i}/\Sigma {x}_{i}{V}_{i}$ where *x*_*i*_, *V*_*i*_, and *E*_*i*_ are the atomic fraction, molar volume, and Young’s modulus of the alloy element *i*. Elemental Young’s modulus values were obtained via Pymatgen^22^.
- **O content (wppm)**: Measured oxygen content.
- **N content (wppm)**: Measured nitrogen content.
- **C content (wppm)**: Measured carbon content.

A portion of the database (the 25 alloys with the highest yield strength at room temperature) is highlighted in Table 1 to provide an example for how data are stored in each field. This is only a subset of the properties collected in the database. Statistics on all properties extracted for the database are presented in Table 2.

**Table 2:** Statistics of the properties captured in the database.

Property	count	unique	mean	std	min	max
Alloy composition	1545	630	—	—	—	—
Processing method	1426	5	—	—	—	—
Microstructure	1402	40	—	—	—	—
grain size (*μ*m)	237	176	90.2	183.0	0.018	2000.0
Exp. Density (g/cm^3^)	112	52	7.6	2.5	1.46	13.6
Calculated Density (g/cm^3^)	1545	82	8.0	1.8	1.4	13.7
Test temperature (°C)	1364	68	228.8	379.1	−268.8	1600.0
HV	530	372	478.3	212.9	94.7	1183.0
YS (MPa)	1067	715	891.0	569.5	24.0	3416.0
UTS (MPa)	535	438	1184.6	721.3	80.0	4023.6
Elongation (%)	619	245	30.2	22.0	0.0	105.0
Elongation plastic (%)	149	85	20.3	25.7	0.0	189.2
Exp. Young modulus (GPa)	145	116	125.0	56.9	16.6	240.0
Calculated Young modulus (GPa)	729	113	166.4	48.9	72.0	298.0
O content (wppm)	57	10	764.0	2085.0	80.0	7946.0
N content (wppm)	45	1	5.0	0.0	5.0	5.0
C content (wppm)	4	4	19911.8	16142.8	1900.0	36380.0

Including mean, standard deviation (std) and the minimum (min) and maximum (max) values.

**Fig. 2:**
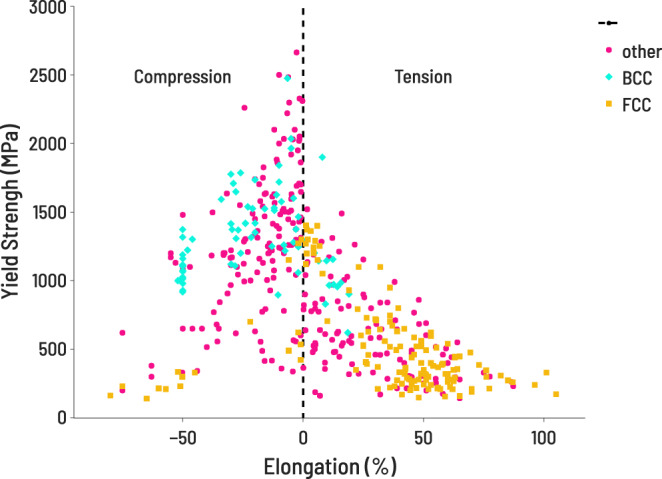
Room-temperature yield strength values plotted against elongation. For visualization purposes, elongation results in compression have been assigned negative values in the plot. Points are colored by structural class (single phase BCC (turquoise), single phase FCC (gold), other (magenta)). “Other” is defined as any report of an MPEA that is either multiphase, or single-phase but not FCC or BCC.

**Fig. 3:**
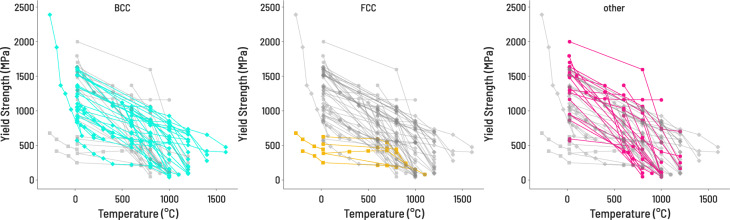
Yield strength as a function of temperature for three classes of HEAs ((**a**) single phase BCC (turquoise), (**b**) single phase FCC (gold), (**c**) other (magenta)). “Other” is defined as any report of an MPEA that is either multiphase, or single-phase but not FCC or BCC. The trend may suggest that BCC MPEAs have better high-temperature strength, but also highlights the lack of data available for FCC MPEAs.

Figure 2 illustrates the relationship between yield strength and elongation for compressive and tensile tests. Figure 3 illustrates the temperature dependence of yield strength across three microstructure classifications (single-phase BCC, single-phase FCC, and multiphase/other).

## Technical Validation

### Review by domain experts

The data were collected, processed, and verified for accuracy by a team familiar with MPEAs and their properties. This domain-knowledge was useful during data compilation and formatting of the dataset.

### Extreme value identification

**Fig. 4:**
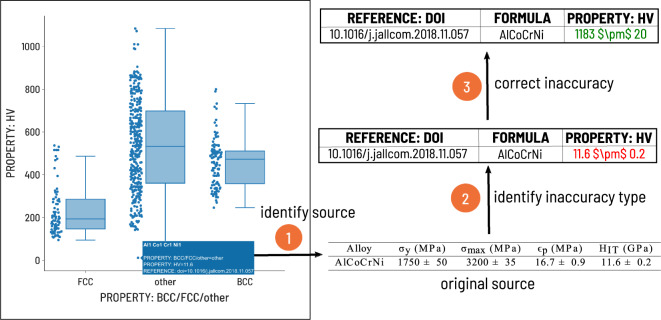
Workflow associated with extreme property value verification and (if necessary) correction. Step 1: Box plots were generated for properties of interest (e.g. alloy hardness) and the source of any extreme values were investigated. Step 2: In this case, the inaccuracy was units-related; the value was recorded as in units of GPa, however the database expected units of HV. Step 3: The value with correct units was updated in place of the originally recorded value. Original source reproduced with data from Jumaev *et al*.^26^.

During the processing of the database, various statistical plots were generated to assist in the identification of outliers and subsequent removal or correction of inaccurate data. Figure 4 is provided as an example of the outlier identification process. Box plots are generated for properties of interest and extreme values in the tails of the distribution are investigated. Extreme values that could not be verified were either removed or corrected.

## Usage Notes

This expanded dataset on MPEAs is intended for use to guide experiment for future alloy development. As shown in Figs. 2 and 3 the dataset can produce informative visualizations to guide researcher efforts. Each record can be accessed programmatically via the Citrination API^23^. In conjunction with traditional Python data processing packages (e.g. pandas) the dataset will be useful as training data for machine learning applications. To ensure data quality, each record is associated with a digital object identifier (DOI) link to the original source. To improve the predictive capabilities of subsequent machine learning models, researchers are encouraged to contribute to this database through the addition of new data as it is generated.

## Data Availability

Data processing, validation and statistical plotting were performed using visualization tools on Citrination and Jupyter notebooks^24^ in a Python 3^25^ environment. The code is available on GitHub (https://github.com/CitrineInformatics/MPEA_dataset).
